# Water Leaching Kinetics of Boron from the Alkali-Activated Ludwigite Ore

**DOI:** 10.3390/molecules29040826

**Published:** 2024-02-12

**Authors:** Binjun Liang, Haixiang Hu, Bin Xiao, Zhigang Lu, Weiquan Yuan, Zheyu Huang

**Affiliations:** 1 School of Resources and Civil Engineering, Gannan University of Science and Technology, Ganzhou 341000, China; liangbinjun1205@163.com (B.L.); hx.hu@gnust.edu.cn (H.H.); lufangang9901@163.com (Z.L.); ywqsdut@163.com (W.Y.); huangzheyu_h@163.com (Z.H.); 2 Ganzhou Key Laboratory of Mine Geological Disaster Prevention and Control and Ecological Restoration, Ganzhou 341000, China

**Keywords:** ludwigite ore, alkali-activated, water leaching, kinetics, diffusion control

## Abstract

The primary aim of this study was to investigate the boron leaching process from alkali-activated ludwigite ore. Initially, the ore underwent activation through roasting at 1050 °C for 60 min with 20% sodium carbonate. Subsequently, the study examined the influence of leaching parameters, including temperature, time, liquid-to-solid ratio, and particle size, using the activated ore as the raw material. Additionally, water leaching characteristics of the residues and boron kinetics were analyzed. The results demonstrated that boron leaching efficiency reached 93.71% from the reduced ludwigite ore under specific conditions: leaching temperature of 180 °C, leaching time of 6 h, liquid-to-solid ratio of 8:1, and feed particle size of 52.31 μm (average particle size). Leach residue characteristics indicated the dissolution of minerals during the process. The boron behavior during water leaching followed the Avrami Equation, and the kinetics equation was derived by fitting the leaching data. Moreover, the activation energy (E_a_) value for boron leaching was determined to be 8.812 kJ·mol^−1^ using the Arrhenius Equation, indicating that the leaching process is controlled by diffusion.

## 1. Introduction

Boron, a crucial rare non-metallic element, falls within the category of strategic mineral resources [1,2]. Widely employed in industry, agriculture, national defense, medicine, nuclear industry, and emerging fields, boron and its compounds possess notable attributes such as light weight, flame retardancy, heat resistance, wear resistance, high hardness, strength, and catalytic properties [3,4,5,6]. Global distribution of boron mineral resources is highly uneven, with approximately 98% concentrated in a handful of countries, notably Turkey (88%), Russia (2.9%), the United States (2.9%), Chile (2.6%), and China (1.8%) [7,8]. Nature hosts approximately 230 types of boron minerals, yet only slightly more than 20 types of ore with B_2_O_3_ contents higher than 12% (lower boron grades being associated with higher production costs)—such as borax, kernite, colemanite, ulexite, borborite, pandermite, hydroboracite, and szaibelyite—are used as raw materials for boron products [9,10,11]. In China, the predominant source of boron raw materials is the limited szaibelyite mineral resource, constituting only 6.64% of the total boron resources of the country [12]. However, the source is nearing depletion after years of exploitation [13]. Fortunately, there is a promising alternative in the form of ludwigite ore, a boron-containing mineral resource with a substantial reserve of 280 million tons located in the Liaoning province of China [14,15]. Despite its potential, ludwigite ore presents challenges, including low boron content, poor boron activity, and complex mineral composition, which impede its direct large-scale development and utilization [16,17]. Addressing these challenges is crucial for maximizing the benefits of this valuable boron resource.

Since the discovery of ludwigite ore, numerous researchers were dedicated to addressing the challenges of boron–iron separation [18,19,20]. One widely recognized and effective approach involved high-temperature smelting. Notable methods included the “blast furnace method”, the “pre-reduction-electric furnace smelting method”, and the “granular iron method” [21,22,23]. The blast furnace method entailed smelting sintered boron-bearing iron concentrate in the blast furnace, resulting in boron-containing pig iron (B content: 0.8–1.2%) and boron-rich slag (B_2_O_3_ content: 12–17%) [21,24]. The pre-reduction electric furnace smelting method involved agglomerating boron-bearing iron concentrate followed by reduction roasting at 850–1000 °C in the coal-based rotary kiln or gas-based vertical furnace to reduce iron oxide to metallic iron. Subsequently, the metallic iron was melted and separated in the electric furnace, resulting in boron-containing molten iron (with the B content of 0.01–0.02%) and boron-rich slag with the B_2_O_3_ content of 21–22% [25]. The granular iron method involved creating carbon-containing pellets from boron-bearing iron concentrate, subjecting them to high-temperature reduction and melting, and obtaining granular iron (B content: 0.065%) and boron-rich slag (B_2_O_3_ content: 20.01%) [22,26]. While these methods yielded high-grade boron-rich slag, they all faced the challenge of low boron activity in the slag. Consequently, the slow cooling process (cooling rate < 2 °C/min within the temperature range of 1200–900 °C) was deemed essential to improve the alkali-dissolution activity of boron. However, the operational challenges associated with this slow cooling approach limited the further utilization of the slag [22,27]. Additionally, the researchers explored a method involving high-temperature synergistic super-gravity field separation for boron–iron, significantly enhancing the separation efficiency of boron and iron [28,29]. Although this method could transfer more than 98% of boron into the slag phase, further research was required to determine whether the activity of the boron-rich slag met the specified requirements. Moreover, the implementation of high-temperature super-gravity separation presented significant challenges in industrial applications.

Currently, a highly effective method for processing ludwigite ore is direct reduction synergistic alkali-activated roasting [30,31]. Numerous research endeavors were undertaken to delve into the intricate mechanisms governing the influence of boron minerals and sodium carbonate in the reduction process of ludwigite ore. These comprehensive investigations aimed to unravel the complexities associated with the roles of boron minerals, such as suanite (roasting product of szaibelyite) or other boron-bearing mineral, and sodium carbonate in shaping and influencing the overarching reduction process of the ludwigite ore. The nuanced interactions at play were meticulously explored to shed light on the specific functions fulfilled by boron minerals and sodium carbonate in fine-tuning the extraction of elemental boron and iron from the ore. The principle involves transforming iron minerals into metallic iron through direct reduction while utilizing the reaction between sodium salt and boron minerals to convert them into soluble borate. The specific reactions are as follows [32,33]:

Direct reduction of iron minerals:

Fe_3_O_4_ + 4CO = 3Fe + 4CO_2_ (1)

Reaction between sodium salt and boron minerals:

Mg_2_(OH) [B_2_O_4_(OH)] + Na_2_CO_3_ = 2NaBO_2_ + 2MgO + H_2_O↑ + CO_2_↑ (2)

(Mg, Fe)_2_Fe[BO_3_]O_2_ + Na_2_CO_3_ + CO → NaBO_2_ + Fe + MgO + CO_2_ (3)

This method could effectively process the iron ore to obtain the desired products: powdery direct reduction iron and sodium metaborate. The actual operation results showed that this process had excellent performance, with both the iron grade and recovery rate exceeding 90%. However, the synchronous grinding–leaching recovery rate of boron was only around 70% [30,34]. Moreover, most existing studies have focused narrowly on examining empirical parameters regarding the roasting reaction mechanism, including roasting temperature, roasting time, and atmospheric conditions [35,36]. There has been little investigation into improving the boron leaching rate and leaching mechanism.

As a result, this study utilized reduced ludwigite ore to systematically investigate boron leaching characteristics and kinetics during aqueous processing. The primary objective was elucidating the leaching mechanism of boron-bearing minerals and devising strategies to control their dissolution. The novelty lies in exploring how parameters like temperature, time, liquid–solid ratio, and particle size impact boron leaching kinetics using alkali-treated ore. Microscopic analysis and kinetic data enabled examining the boron leaching mechanism and establishing a quantitative process model. Notably, high temperature sodium carbonate activation was creatively employed to induce mineral phase transformations enhancing boron leachability. Through investigating the dissolution behaviors of roasted boron iron minerals, efficient boron extraction was successfully achieved, which will provide guidance for effective and sustainable development of boron iron ore resources. In summary, this was a systematic investigation uncovering boron leaching kinetics from ludwigite ore and devising optimized extraction parameters and efficiency through elucidation of the mechanism. This work provides an enhanced methodology and framework for optimizing boron extraction from complex ores that can inform and enlighten other researchers exploring novel leaching processes.

## 2. Results and Discussion

### 2.1. Effect of Water Leaching Parameters on Boron Leaching

In this section, the influence of leaching parameters such as leaching temperature, liquid–solid ratio, and particle size of raw materials on boron leaching under different leaching times was investigated. These parameters were specially selected because they were key operating variables controlling boron extraction efficiency. The influence of these parameters could be clarified through systematic parameter study, and the optimized conditions for improving the leaching kinetics and recovery rate of boron could be determined.

#### 2.1.1. Effect of Leaching Temperature on Boron Leaching

**Figure 1:**
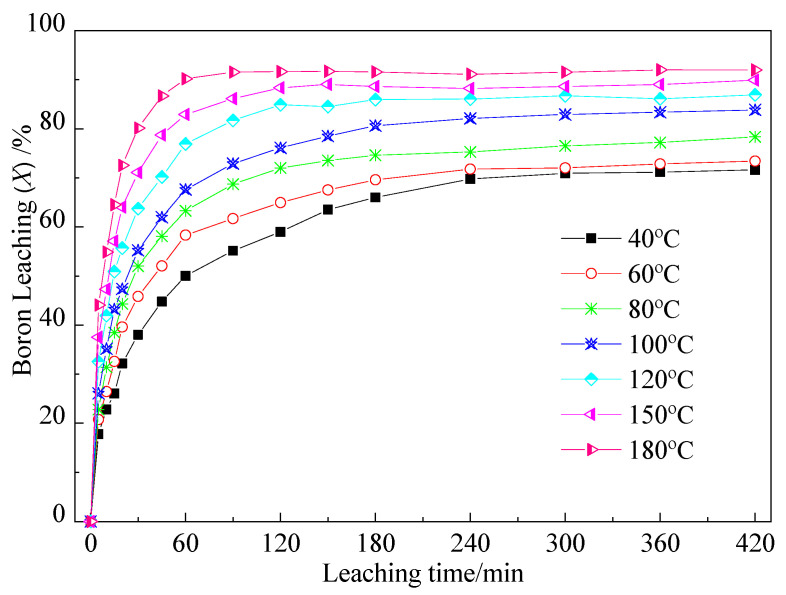
Effects of leaching temperature on boron leaching under the following conditions: liquid-to-solid ratio of 10:1 and average feed particle size of 60.16 μm.

The effects of leaching temperature on boron leaching were investigated at various leaching times, maintaining the liquid-to-solid ratio of 10:1 and the average feed particle size of 60.16 μm. The results are illustrated in Figure 1. It was observed that both leaching temperature and leaching time played pivotal roles in the water leaching process of boron. At lower temperatures, such as 40 °C and 60 °C, the leaching rates of boron were relatively low, measuring only 72.01% and 72.39%, respectively, after 6 h of leaching. However, as anticipated, the leaching rate exhibited a significant increase with temperature. Within the high-temperature range of 150–180 °C, the boron leaching rate reached approximately 90% after 60 min of leaching, escalating to 92.01% after 6 h of leaching at 180 °C. In contrast, under low-temperature conditions (40–80 °C), boron was found to be completely unextractable from the activated ore. This phenomenon is likely attributed to the encapsulation of a portion of sodium borate within other minerals.

#### 2.1.2. Effect of Liquid-to-Solid Ratio on Boron Leaching

**Figure 2:**
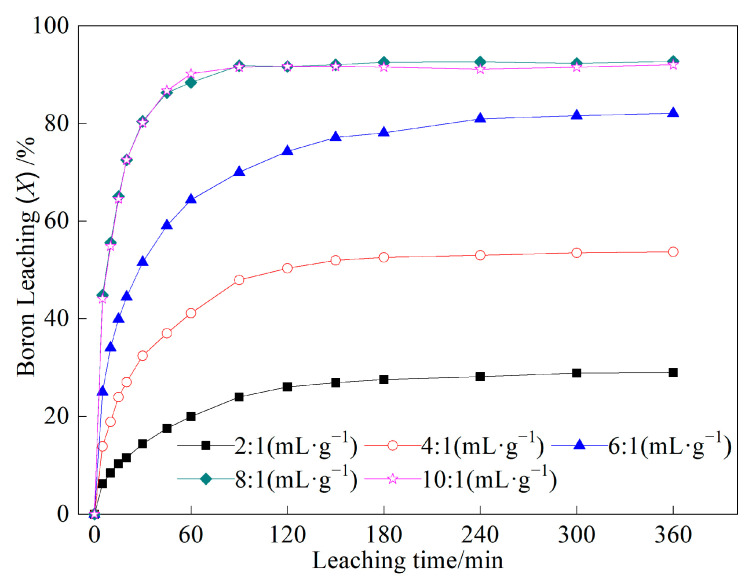
Effects of liquid-to-solid ratio on the boron leaching under the leaching conditions of leaching temperature of 180 °C and average feed particle size of 60.16 μm.

The effects of the liquid-to-solid ratio on boron leaching were investigated at a leaching temperature of 180 °C and an average feed particle size of 60.16 μm. As depicted in Figure 2, the results clearly indicated that boron leaching varied significantly with changes in the liquid-to-solid ratio. Upon analyzing Figure 2, it became apparent that when the liquid-to-solid ratio was below 6:1, there was a notable increase in boron leaching with the rising liquid-to-solid ratio. This observation suggested that the higher liquid-to-solid ratio facilitated more efficient leaching of boron from the ore particles. Furthermore, as the liquid-to-solid ratio was further increased to 8:1, the boron leaching essentially reached its maximum value under the same leaching conditions. This suggested that beyond this ratio, increasing the liquid-to-solid ratio did not yield significant improvements in boron leaching. The experimental results also supported this observation; when the liquid-solid ratio was increased to 10:1, no further increase in boron leaching was observed. This implied that further increasing the ratio did not provide additional benefits and, conversely, increased the workload without commensurate improvement.

#### 2.1.3. Effect of Feed Particle Size on Boron Leaching

**Figure 3:**
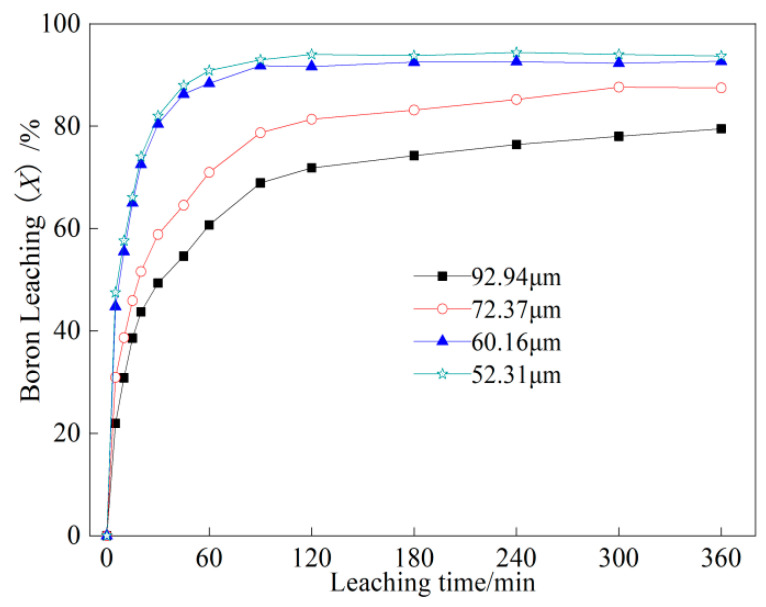
Effects of feed particle size on boron leaching under the following conditions: leaching temperature of 180 °C and liquid-to-solid ratio of 8:1.

The study explored the effects of the average feed particle size on boron leaching while maintaining the leaching temperature of 180 °C and the liquid-to-solid ratio of 8:1. The particle size of the reduced ore was determined at the different sampling times (5 s, 10 s, 15 s, and 20 s), resulting in corresponding average particle sizes of 92.94 μm, 72.37 μm, 60.16 μm, and 52.31 μm, respectively. The leaching experiment results for different particle sizes were presented in Figure 3. The diagram distinctly illustrated that the feed particle size also significantly influenced boron leaching. The smaller particle sizes led to the higher leaching rates at the same leaching time. This was attributed to smaller ore particles having higher specific surface area, exposing more borate to the leaching solution. Furthermore, as the leaching process progressed, the solution could penetrate into micro-cracks, facilitating more effective dissolution of the borate.

Based on the findings, the most favorable conditions for boron leaching were a leaching temperature of 180 °C, a leaching time of 6 h, a liquid-solid ratio of 8:1, and an average feed size of 52.31 μm. The optimized conditions ensured maximum borate dissolution, with the leaching rate reaching the maximum value of 93.71%.

### 2.2. Characterization of Leach Residues

**Figure 4:**
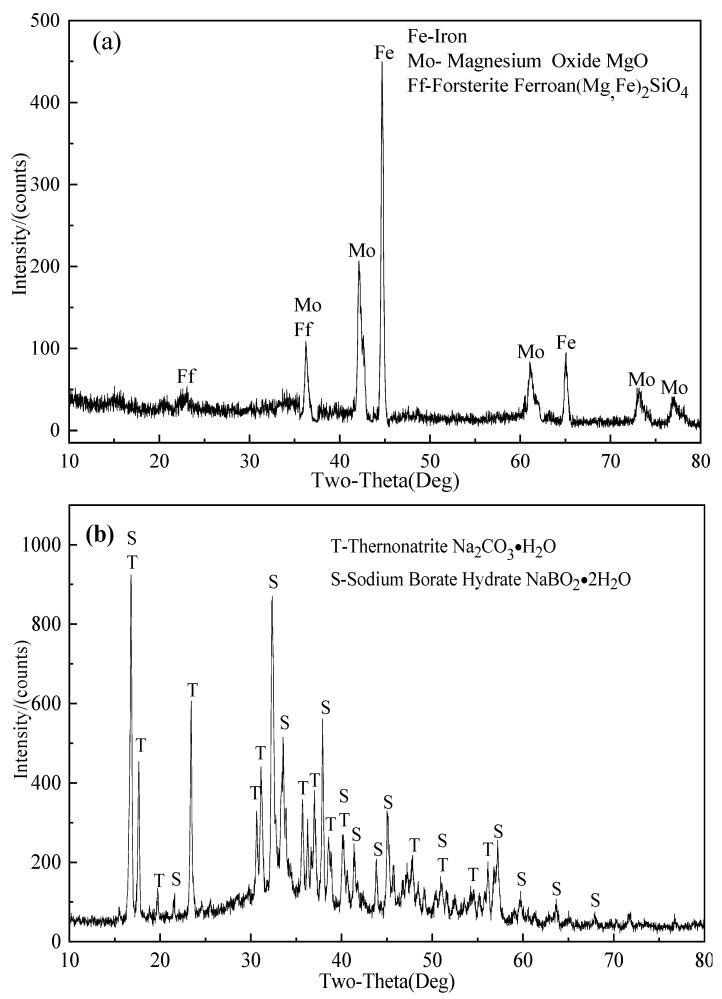
XRD patterns of the products obtained under the following leaching conditions: temperature of 180 °C, time of 6 h, liquid-to-solid ratio of 8:1, and average feed particle size of 60.16 μm. (**a**) Leaching residue, (**b**) evaporation–crystallization product of the water leaching liquor.

**Figure 5:**
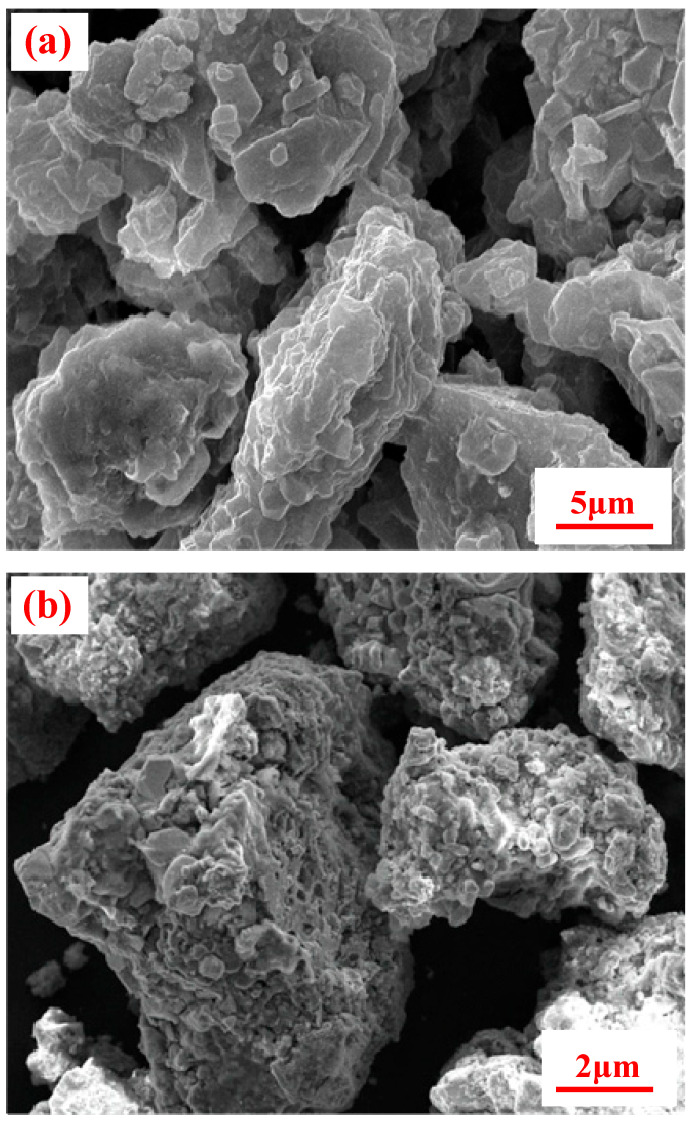
SEM images of activated ore and residues ((**a**) activated ore, magnification 2000 times; (**b**) residue of leaching at 180 °C for 60 min with liquid-to-solid ratio of 8:1, magnification 5000 times).

In Figure 4, the XRD patterns of the products obtained from the leached sample were presented. When comparing Figure 5 with Figure 1, it was evident that the sodium-containing compounds had significantly disappeared after the leaching process (Figure 4a), indicating their dissolution. This dissolution phenomenon demonstrated that the leaching process effectively extracted these compounds. Additionally, the analysis of the evaporative crystalline product from the leachate (Figure 4b) confirmed the presence of sodium metaborate during the roasting process. This finding provided solid evidence that szaibelyite reacted with sodium carbonate (Equations (2) and (3)) under high-temperature conditions. Sodium metaborate is known for its high solubility and versatile applications [37]. As a result, the reactions were considered the primary reason responsible for the enhanced boron activity.

Simultaneously, the scanning electron microscopy (SEM) was employed to observe the particle morphology before and after the leaching process, as depicted in Figure 5. Figure 5a illustrated the surface of the un-leached reduced ore particles, which appeared relatively flat and smooth. However, after leaching, the surface of the reduced ore particles no longer exhibited the flat and smooth appearance. Instead, it displayed varying degrees of unevenness and even the formation of holes (Figure 5b). This alteration in particle morphology provided additional evidence that some certain substances dissolved during the water leaching process. The dissolution of these substances contributed to the modified morphology, as the removal of dissolved materials could create voids or irregularities on the particle surface. Moreover, this observation complemented the earlier-discussed XRD analysis and further confirmed the presence of sodium-bearing compounds. This finding added more evidence to support the notion that certain minerals were converted into soluble sodium salts during the reductive roasting.

**Figure 6:**
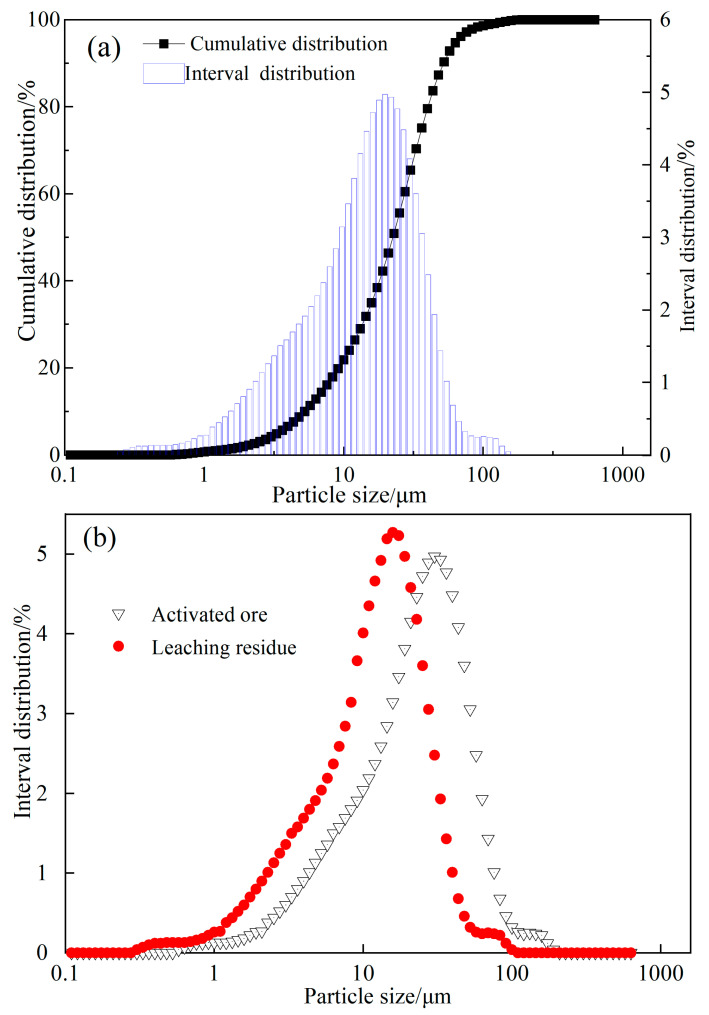
Particle size distributions of activated ore and the water leaching residue ((**a**) cumulative distribution curve of particle size of activated ore, (**b**) particle size distribution of activated ore and leaching residue).

Laser particle size analysis was also conducted on the reduced ore samples before and after leaching, and the results are presented in Figure 6. Figure 6a illustrated the cumulative particle size distribution of the reduced ore prior to leaching (sample preparation time: 15 s) with the cumulative distribution of particles smaller than 80.96 μm accounting for 97.17% and the average particle size of 60.16 μm. Comparing the volume distribution of the particle sizes before and after leaching of the reduced ore (Figure 6b), it is evident that the average particle size of the reduced ore decreases after water leaching, with the average particle size after leaching measuring only 55.83 μm. As the particle size decreased, the presence of soluble substances was further confirmed.

### 2.3. Kinetics of Boron Water Leaching

#### 2.3.1. Kinetic Model Selection

The objective of studying the kinetics of the leaching process was to determine the factors influencing leaching velocity and identify the controlling steps of the leaching process. This understanding is crucial for enhancing the leaching efficiency and improving leaching indicators. The frequently used kinetic model for the leaching process is the shrinking core model, typically applicable when the particle size of the leaching material falls within a narrow range (e.g., −74 μm to +38 μm or −38 μm) [38,39]. However, in this study, the particle size range of the material was wide (0–74 μm), rendering it unsuitable for description by this model. Therefore, the investigation sought a more suitable kinetic model to better capture the characteristics of the leaching process under the specific conditions of this study.

In Section 2.2, it was detailed that the continuous dissolution of minerals not only resulted in the formation of pores on particle surfaces but also led to the reduction in particle size as the leaching process unfolded. Pores emerged, and the decrease in particle size was primarily attributed to the dissolution of soluble substances, particularly sodium salts. This dynamic transformation highlighted the evolving microstructure of the material during the leaching process. Furthermore, the results of the leaching tests revealed the distinctive dissolution characteristics of activated ores, characterized by the substantial initial dissolution rate followed by the gradual decline as the leaching time increased. The observation also implied the nuanced interplay among factors influencing the kinetics of solid–liquid reactions. The Avrami Equation served as a valuable tool in understanding the kinetics of the intricate solid–liquid reactions [40,41]. Initially employed to study nucleation and growth kinetics in multiphase chemical reactions, the Avrami Equation found subsequent applications in analyzing kinetics in metal and metal oxide leaching processes [42,43,44]. The equation provided a comprehensive framework for understanding the evolutionary nature of certain leaching processes and offer insights into describing the kinetic behavior of leaching processes. The general expression of the equation is as follows:

(4) $$\lambda =1-\exp(-k\cdot t^{n})=1-e^{-kt^{n}}$$

where λ is the extent of the reaction (i.e., leaching yield), k is the rate constant of the leaching reaction, t is the leaching time, and n is the mineral grain property and geometric shape function, which remains invariant under leaching conditions.

The Avrami equation characterize leaching kinetics by describing reactions with different reaction orders represented by the n value, providing insights into how the reaction rate evolves over time: when n is less than 1, it indicates an initially fast rate that diminishes as dissolution becomes diffusion-limited (This feature aligns well with the leaching characteristics observed in our study); when n equals 1, it represents a surface-reaction controlled process with a constant initial rate; when n exceeds 1, it fits phenomena of an induction period caused by precipitate formation or other effects with a slow initial rate accelerating over time [45,46,47]. Fitting this equation to data yields quantitative kinetic parameters comprising the n value and rate constant that delineate the time-dependence of the leaching reaction, enabling elucidation of the rate-limiting steps and analysis of how variables like temperature, acid concentration, and particle size impact the kinetics to ultimately interpret the governing leaching mechanism.

#### 2.3.2. Discussion and Analysis of Kinetics

By taking the natural logarithm of both sides of Equation (4), we derived the following equation:

(5) ln[−ln(1−λ)]=lnk+nlnt

**Figure 7:**
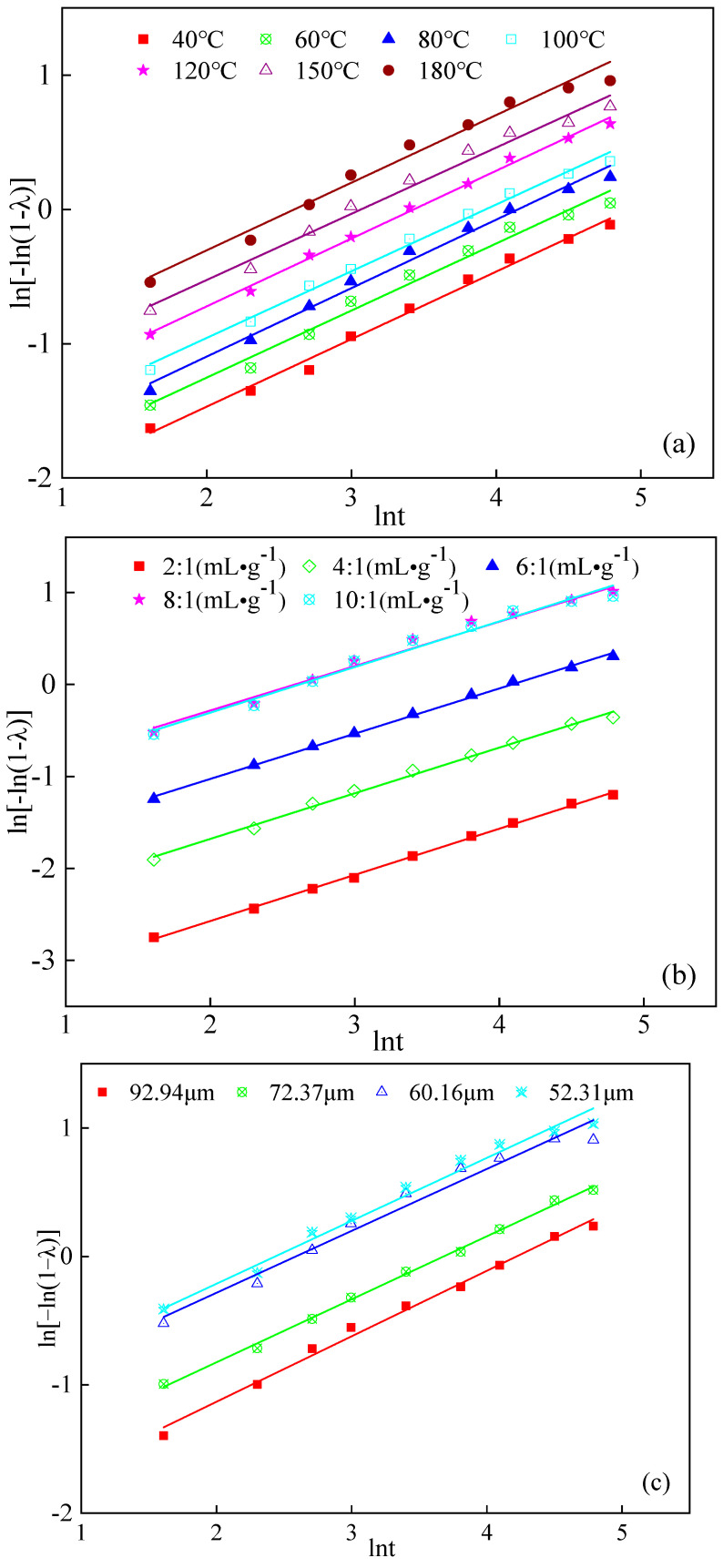
Plots of ln[−ln(1 − λ)] vs. lnt for leaching parameters (**a**) different temperatures; (**b**) liquid-to-solid ratios; (**c**) average particle size.

Then, by substituting the experimental data from Figure 1, Figure 2 and Figure 3 into Equation (5), we obtained ln[−ln(1 − λ)] to lnt plots as shown in Figure 7. Furthermore, fitting the data under different leaching conditions allowed us to determine the values of n, lnk, and the coefficient of linear correlation R^2^. All the obtained values are listed in Table 1.

To clarify, in the fitting results, the parameter n represented the slope of the fitted line and lnk represented the intercept. The coefficient of linear correlation R^2^ indicated the goodness of fit between the experimental data and the fitted curve. The higher R^2^ value suggested the better fit, indicating that the model accurately represented the leaching process. Moreover, the values of n and lnk provided insights into the reaction kinetics and the rate of leaching, and it can be seen from Table 1 that the linear correlation coefficients of the data fitting under various leaching conditions were all above 97%. The high correlations indicated that the kinetics of boron leaching follow the Avrami Equation. Additionally, we obtained n = 0.50 by averaging the fitted values of n, which suggested that the grain properties of sodium borate during the water leaching process were characterized by the value of 0.50. The value of n being less than 1 also confirmed that the Avrami Equation was consistent with the leaching properties in our present study.

#### 2.3.3. Derivation of the Kinetic Equation

If the leaching time remained constant, the parameters influencing boron leaching were the leaching temperature, the liquid-to-solid ratio, and the feed particle size. Consequently, the kinetic equation could be formulated as follows:

(6) $$k=A_{0}(e^{\frac{-E_{a}}{\mathrm{RT}}}\cdot L_{0}^{a}\cdot r_{0}^{b})$$

where k is the leaching rate constant (s^−1^); E_a_ is the apparent activation energy (J·mol^−1^); T is the leaching temperature (K); L_0_ is the liquid-to-solid ratio (mL·g^−1^); r_0_ is the average particle size of the leaching material (μm); and A_0_, a, and b are undetermined parameters.

By taking the logarithm of both sides of Equation (6), we derived Equation (7) as follows:

(7) $$\mathrm{lnk}={\mathrm{lnA}}_{0}+\frac{-E_{a}}{\mathrm{RT}}+{\mathrm{alnL}}_{0}+{\mathrm{blnr}}_{0}$$

**Figure 8:**
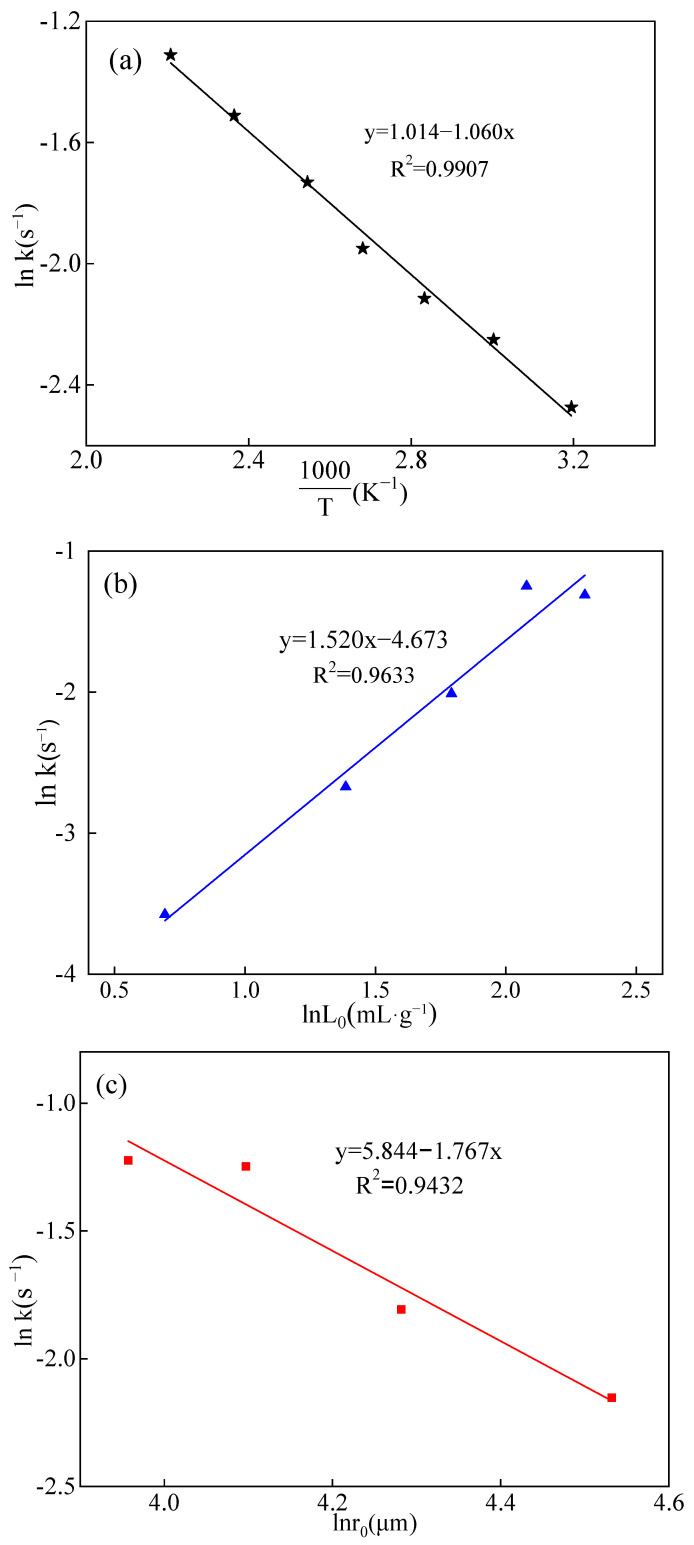
Plot of lnk vs. $\frac{1}{T}$, lnL_0_, and lnr_0_, lnL_0_, and lnr_0_ : (**a**) lnk vs. $\frac{1}{T}$; (**b**) lnk vs. lnL_0_; (**c**) lnk vs. lnr_0_.

Then, the lnk was plotted against $\frac{1}{T}$, lnL_0_ and lnr_0_, respectively, and the results were shown in Figure 8. The Figure 8 clearly illustrated the correlation between the leaching rate of boron and the leaching temperature, the liquid-to-solid ratio, and the feed particle size. It was evident that the leaching rate of boron exhibited a direct relationship with both the temperature and the liquid-to-solid ratio, indicating that the leaching rate increased with higher temperatures and greater liquid-to-solid ratios. Conversely, the leaching rate of boron demonstrated an inverse relationship with the feed particle size, meaning that the leaching rate of boron increased as the particle size decreased.

Meanwhile, the relationship between the apparent reaction rate constant k and the absolute temperature T satisfied the Arrhenius Equation at different temperatures [48]. The formula is shown as Equation (8):

(8) $$k=A\cdot e^{\frac{-E_{a}}{\mathrm{RT}}}$$

where A is the pre-exponential factor (L·mol^−1^·s^−1^), E_a_ is the apparent activation energy (kJ·mol^−1^), and R is the universal gas constant (8.314 J·K^−1^·mol^−1^).

To calculate the pre-exponential factor A and activation energy E_a_, taking the logarithm of both sides of Equation (8) gives:

(9) $$\mathrm{lnk}=-\frac{E_{a}}{\mathrm{RT}}+\mathrm{lnA}$$

In Equation (9), $-\frac{E_{a}}{R}$ and lnA correspond to the slope and intercept in Figure 8a, respectively. By substituting the slope value of −1.060 for $-\frac{E_{a}}{R}$ and the intercept value of 1.014 for lnA, the activation energy E_a_ and pre-exponential factor A were calculated as 8.812 kJ·mol^−1^ and 2.757, respectively. Similarly, the slopes of 1.520 and −1.767 in Figure 8b,c represent the values of parameters a and b, respectively, in Equation (7), while the sum of all intercepts (2.185) in Figure 8 corresponds to the value of lnA_0_ in Equation (7). Furthermore, with lnA_0_ = 2.185, A_0_ was calculated to be 8.891. Hence, Equation (6) could be rewritten as follows:

(10) $$k=8.891(e^{\frac{-8812}{\mathrm{RT}}}\cdot L_{0}^{1.520}\cdot r_{0}^{-1.767})$$

Then, the water-leaching kinetics equation of boron could be expressed as Equation (11) combined with Equation (4) and Equation (10). The specific representation of the equation is as follows:

(11) $$\lambda =1-\exp\{8.891[\exp(\frac{-8812}{\mathrm{RT}})\cdot L_{0}^{1.520}\cdot r_{0}^{-1.767}]\cdot t^{0.5}\}$$

## 3. Materials and Methods

### 3.1. Materials

**Figure 9:**
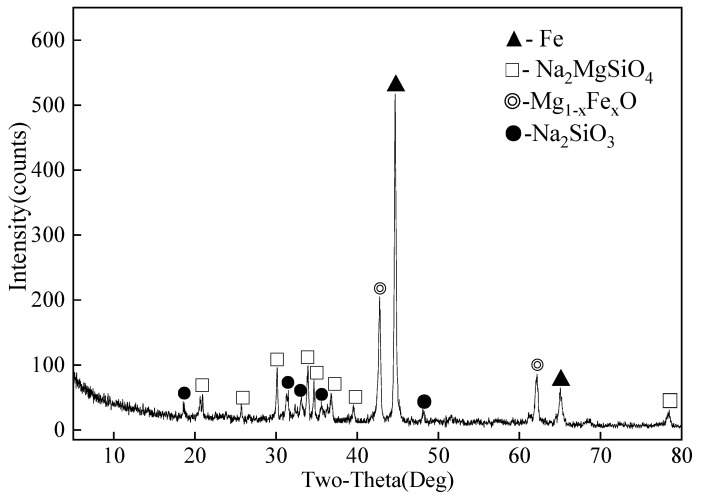
XRD patterns of reduced ludwigite ore roasting at 1050 °C for 60 min in the presence of 20% Na_2_CO_3_.

The comprehensive properties of ludwigite ore, including its chemical composition and phase analysis, were detailed in our previous works [30,33]. Building upon the insights derived from our research, the optimal conditions for ore activation roasting were established at the reduction temperature of 1050 °C for 60 min in the presence of 20% Na_2_CO_3_ [32]. The raw materials employed in this study comprise the roasting products obtained under the aforementioned conditions. Detailed chemical analysis is available in Table 2, where component content was determined by chemical titration. XRD analysis is presented in Figure 9. The XRD results indicate that the reduced ludwigite ore primarily consists of metallic iron (Fe) and compounds such as sodium magnesium silicate (Na_2_MgSiO_4_), sodium silicate (Na_2_SiO_3_), and magnesium ferrous oxide (Mg_1−x_FexO). Notably, the diagram does not exhibit the presence of sodium metaborate, which may be attributed to the calcination temperature (1050 °C) surpassing its melting point (966 °C), resulting in the formation of amorphous presence of sodium metaborate.

### 3.2. Methods

#### 3.2.1. Alkali-Activated Ludwigite Ore Process

The process involves thoroughly mixing the boron iron concentrate with sodium carbonate, where the sodium carbonate is added at 20% of the mass of the boron–iron concentrate, and forming the mixture into pellets for subsequent drying. These dried pellets undergo reduction roasting at 1050 °C for 60 min. Then, the cooled pellets are milled post-cooling to achieve a grind size with complete 0.074 mm undersize for leaching requirements alongside derivation of an as-ground subsample for XRD examination. The XRD patterns are generated using an X-ray diffractometer (Rigaku D/max-2500, Rigaku Corporation, Tokyo, Japan) with the Cu anode target under the following conditions: radiation tube voltage 40 kV, tube current 250 mA, incident slit 1°, anti-scattering slit 1°, receiving slit 0.45°, Soller slit 0.3 mm, wavelength 1.54 Å, step size 0.02°, scanning speed of 8°/min, and scanning range from 10° to 80°.

#### 3.2.2. Water Leaching Process of Boron

The boron leaching experiments were conducted utilizing the DY-8 autoclave. Self-made equipment of Central South University, Changsha, China). Specific details on the leaching process and the calculation of the leaching rate are not provided in this paper. For more specific information, please refer to our previous research [32].

#### 3.2.3. Analysis of Ore Particle Morphology

In this study, the JSM-6360 scanning electron microscope (Japan Electronics Co., Ltd., Tokyo, Japan) was employed to observe the morphological characteristics of the ore particles under various leaching conditions. The specific instrument parameters were set as follows: the maximum magnification was 100,000 times (during the experiments, we determined appropriate magnification ratios contingent on the requisite clarity detail needs), the minimum resolution was 3 nm, and the spectrum measurement range covered all elements except H, He, Li, and Be.

#### 3.2.4. Particle Size Analysis of Ore

The Bettersize 2600E (Dandong Baxter Instrument Co., LTD, Dandong, China) laser particle size analyzer was employed to determine the average particle size (using D_50_ as the representative value) of the materials. The measurement conditions were set as follows: measurement range: 0.1–1500 μm; resolution ratio: A-level (UF1211-2008, multimodal); repeatability and accuracy error ≤ 1% (deviation of national standard sample D_50_); circulation system: water flow rate 3000–8000 mL/min; number of detectors: 90.

## 4. Conclusions

After conducting the thorough investigation into the influences of boron leaching from the alkali-activated ludwigite ore and the water leaching kinetics of boron, this study provided valuable insights for improving and controlling the efficiency of boron leaching. The main conclusions were outlined as follows:

(1) The extraction of boron from the ludwigite ore demonstrated remarkable efficiency under specific conditions. Specifically, the ludwigite ore underwent the reduction process at 1050 °C for 60 min in the presence of 20% sodium carbonate. Subsequently, the impressive boron extraction rate of 93.71% was achieved through water leaching at 180 °C for 6 h, with a liquid-to-solid ratio of 8:1 and an average feed particle size of 52.31 μm. These specific parameters illustrated a successful method for optimizing boron extraction from the ludwigite ore.
(2) Optimizing boron leaching efficiency requires careful control of key parameters including temperature, leaching time, liquid-to-solid ratio, and ore particle size. Higher temperatures accelerate reactions and dissolution; prolonged times allow complete mineral dissolution; higher liquid-to-solid ratios increase solution–particle contact; and smaller particle sizes increase surface area and solution interaction. Precisely optimizing these parameters is essential for effective and sustainable boron extraction.
(3) The study utilized the Avrami Equation to characterize the water leaching kinetics of boron and established the kinetics equation by fitting the experimental data; the equation could be expressed as: $\lambda =1-\exp\{8.891[\exp(\frac{-8812}{\mathrm{RT}})•L_{0}^{1.520}•r_{0}^{-1.767}]•t^{0.5}\}$.
(4) This study applied the Arrhenius Equation to ascertain the activation energy (E_a_) of 8.812 kJ·mol^−1^ for the water leaching of boron. The finding suggested that the boron leaching process occurred within the diffusion control range, affirming the nature of the leaching process as the solvation reaction.

## Figures and Tables

**Table 1:** Fitting results of linear equation at different leaching conditions.

Leaching Conditions		Correlation Coefficient/R^2^	n	lnk
Temperature (°C)	40	0.9923	0.5027	−2.4738
	60	0.9849	0.4993	−2.2507
	80	0.9902	0.5096	−2.1147
	100	0.9938	0.4966	−1.9498
	120	0.9964	0.5049	−1.7313
	150	0.9854	0.4929	−1.5112
	180	0.9789	0.5034	−1.3110
Liquid-to-solid ratio (mL·g^−1^)	2:1	0.9983	0.5019	−3.5755
	4:1	0.9946	0.4962	−2.6715
	6:1	0.9978	0.4917	−2.0104
	8:1	0.9701	0.4825	−1.2481
	10:1	0.9789	0.5034	−1.3110
Particle size (μm)	92.94	0.9916	0.5103	−2.1528
	72.37	0.9972	0.4911	−1.8071
	60.16	0.9701	0.4825	−1.2481
	52.31	0.9792	0.4959	−1.2240

**Table 2:** Main chemical composition of the reduced ludwigite ore sample/wt.%.

TFe *	FeO	B_2_O_3_	Na_2_O	SiO_2_	MgO	Al_2_O_3_	CaO	P	S
58.23	5.25	5.12	7.22	6.18	14.05	0.43	0.68	0.014	1.12

* The term “TFe” represents the total iron content, which is equal to the sum of the ferrous and the metallic iron contents.

## Data Availability

The data that support the findings of this study are available from the corresponding author upon reasonable request.
